# Evaluation of the Effect of Antibacterial Peptides on Model Monolayers

**DOI:** 10.3390/ijms241914861

**Published:** 2023-10-03

**Authors:** Iwona Golonka, Jakub E. Pucułek, Katarzyna E. Greber, Andrzej Dryś, Wiesław Sawicki, Witold Musiał

**Affiliations:** 1 Department of Physical Chemistry and Biophysics, Wroclaw Medical University, Borowska 211A, 50–556 Wrocław, Poland; iwona.golonka@umw.edu.pl (I.G.); jakub.puculek@onet.pl (J.E.P.); andrzej.drys@umw.edu.pl (A.D.); 2 Department of Physical Chemistry, Faculty of Pharmacy, Medical University of Gdańsk, Al. Gen. J. Hallera 107, 80-416 Gdańsk, Poland; katarzyna.greber@gumed.edu.pl (K.E.G.); wieslaw.sawicki@gumed.edu.pl (W.S.)

**Keywords:** Langmuir monolayer, π–A isotherms, compression reversibility factor, peptides, model biological membrane

## Abstract

The aim of the study was to assess the effect of the synthesized antibacterial peptides: P2 (WKWK)_2_-KWKWK-NH_2_, P4 (C12)_2_-KKKK-NH_2_, P5 (KWK)_2_-KWWW-NH_2_, and P6 (KK)_2_-KWWW-NH_2_ on the physicochemical properties of a model biological membrane made of azolectin or lecithin. The Langmuir Wilhelmy method was used for the experiments. Based on the compressibility factor, it was determined that the monolayers formed of azolectin and peptides in the aqueous subphase are in the condensed liquid phase. At the boundary between the condensed and expanded liquid phases, there was a monolayer made of lecithin and P4, P5 or P6 in the aqueous subphase. In turn, the film consisting of lecithin alone (37.7 mN/m) and lecithin and P2 (42.6 mN/m) in the water subphase was in the expanded liquid phase. All peptides change, to varying degrees, the organization and packing of molecules in the monolayer, both those made of azolectin and of lecithin. The test results can be used for further research to design a system with the expected properties for specific organisms.

## 1. Introduction

In recent years, numerous model biological membranes were developed and studied. Both the single-layer and double-layer models are currently used in various research fields, which emphasizes their high usefulness in the exploration of the natural world [1]. Since the properties of model systems change significantly in the presence of amino acid compounds, it is reasonable to extend and systematize the existing knowledge on the interaction of various antimicrobial peptides with model membranes [2,3,4]. Thanks to their proven antibacterial properties, surfactants consisting of amino acid residues are a promising group that can help combat antibiotic resistance in the near future [5]. The molecular mass of antimicrobial peptides varies depending on whether they are of natural or synthetic origin. The naturally environmental peptides usually contain more than fifteen amino acid residues per molecule, whereas the synthetic ones count less than ten amino acid residues [6]. The ratio of cationic and hydrophobic residues determines the activity of AMPs (antimicrobial peptides). The cationic peptides include such amino acids as arginine (R), lysine (K) or histidine (H), which mediate interactions with negatively charged bacterial lipids. Hydrophobic residues containing tryptophan—W, phenylalanine—F, and leucine—L are also involved in connecting with the cell membrane and influence its damage [7,8]. The cationic functionals facilitate the solubilization of molecules in water, while the lipophilic groups enable localization in lipid micelles [9,10]. Natural AMPs are produced by an entire spectrum of life forms, from prokaryotes to humans. Their antimicrobial activity against pathogens, including Gram-positive and Gram-negative bacteria, viruses and fungi, proceeds through various mechanisms, for example, through membrane disruption, affecting intracellular structures or immunomodulation [11,12].

Short synthesized cationic lipopeptides are an attractive alternative compared to longer natural antimicrobial peptides due to their simple structure, greater physicochemical stability resulting from the presence of D-amino acids and fatty acids, or C-terminal amidation. In addition, short cationic lipopeptides are amphiphilic molecules, so they tend to form aggregates (micelles) with a hydrophobic core and a hydrophilic surface. As a result, they are better protected against proteolytic degradation compared to non-aggregating peptides [13,14].

The Langmuir technique is a method used to study model cell membranes, which reflect the first barrier to drugs applied against numerous microorganisms [15]. These are complex structures with diverse compositions, but the main fraction is made up of phospholipids [16]. Simplified biomimetric systems are useful to study interactions at the molecular level and to analyze the effect of a specific components on the membranes. It allows for the controlling of the composition of lipids, molecular packing, physical states, and experimental conditions such as temperature. Phospholipid monolayers formed using the Langmuir technique are two-dimensional asymmetric structures, have a flat geometry, and may be used to study the processes on the membrane surface [17,18]. Langmuir monolayers have the unique advantage of being able to obtain different densities and compositions of lipids at the interface in a controlled manner, thanks to which the energetics can be studied using surface tension measurements. Water molecules are attracted to the bulk subphase, which generates surface tension. The placement of an amphiphilic compound on the aqueous surface influences the surface tension, which provides information about lipid–lipid and lipid–water interactions. The Langmuir monolayer technique enables the formation of a lipid film on the aqueous subphase and the characterization of lipid–lipid, lipid–water or lipid–drug interactions based on compression isotherms obtained by measuring the surface pressure (π) of the monolayer interfacial as a function of the mean molecular surface area (A). Physicochemical properties, i.e., the compressibility, state of aggregation, and surface occupied by a molecule, of a Langmuir monolayer made of phospholipid molecules compressed to a surface pressure of 30–35 mN/m are comparable to those found in natural cell membranes [19]. From the research we have published so far, we know that the following peptides: P1 (WK)_2_-KWK-NH_2_, P2 (WKWK)_2_-KWKWK-NH_2_, P3 (WR)_2_-KWR-NH_2_, P4 (C12)_2_-KKKK-NH_2_, P5 (KWK)_2_-KWWW- NH_2_, and P6 (KK)_2_-KWWW-NH_2_ exhibit antioxidant properties, whereas the P2, P4, P5, and P6 compounds exhibit antimicrobial activity against S. aureus. Peptide P2 is highly effective against S. aureus. The sorption of P2 and P4–P6 on the polymer—bacterial cellulose (BC) produced by Komagateibacter xylinu confirmed the prospective topical application of these peptides on the BC carrier. The mentioned compounds had no cytotoxic activity against fibroblast lines [20]. So far, only peptide P4 has been shown to have antifungal activity against Candida albicans, Candida tropicalis, and Aspergillus niger, and has antibacterial activity against gram-positive Staphylococcus epidermidis, Bacillus subtilis, and Enterococcus faecalis, as well as against gram-negative Escherichia coli, Klebsiella pneumonia, and Pseudomonas aeruginosa [21]. Four of the six mentioned peptides were selected for further research: antibacterial P2, P4, P5, and P6. One of our hypotheses was that these peptides interfere with bacterial membrane integrity. Therefore, the Langmuir method was chosen as the main research method, to observe the behavior of peptides in the aqueous subphase against the monolayer made of azolectin and lecithin as model cell membranes. Azolectin is a dry soybean extract that contains approximately equal proportions of lecithin, cephalin, and phosphatidylinositol along with small amounts of other phospholipids and polar lipids [22]. Lecithin is a general term used to describe a multi-component mixture of lipids: triglycerides, fatty acids, sterols, glycolipids, and phospholipids, which are the structural and functional components of a variety of cell membranes in plants as well as in various organisms [23,24].

The aim of the study was to assess the impact of the synthesized peptides: P2, P4-P6 on the physicochemical properties of the model monolayer formed from azolectin or lecithin.

## 2. Results

### 2.1. Monolayer Compression Isotherms of the Monolayer Formed from Azolectin with Peptides in the Aqueous Subphase

**Figure 1:**
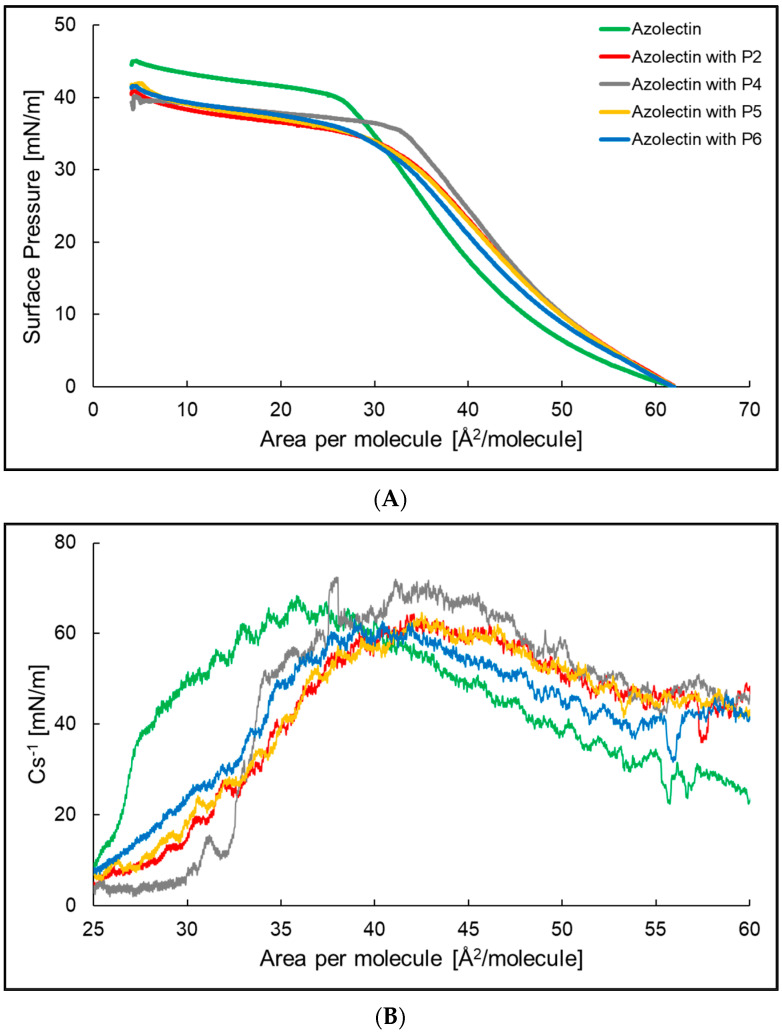
The compression isotherms (**A**) and dependence of the compressibility coefficient depending on the surface area per molecule (**B**) of the azolectin monolayer (─) at the air/water interface at T = 25 °C with P2 (─), P4 (─), P5 (─), and P6 (─) in the water subphase.

The basis for the analysis of the interactions between components within the monolayer, as well as the components forming the film and present in the subphase, is the dependence of changes in surface pressure (π) as a function of surface area per molecule in the monolayer, (A) surface pressure, defined as the difference between the surface tension of water with and without the presence of the Langmuir film, was measured using the Wilhelmy plate method. During this process, the state of the monolayer changes between the gaseous (G), expanded liquid (LE), condensed liquid (LC), and solid (S) phases, accompanied by intermolecular interactions. Specific states correspond to specific orientations and packing of molecules on the surface of the subphase. Therefore, the analysis of the shape, course, and position of the π-A plots provides information about phase transformations, its state, organization, and stability, including the packing density, orientation, and conformation of monolayer molecules. Initially, compression isotherms of azolectin monolayer on the aqueous subphase were performed, as a reference for other systems, composed of azolectin and aqueous solutions of P2, P3, P4, and P5 peptides successively placed in the aqueous subphase, at 25 °C—Figure 1A. The addition of P2, P4–P6 peptides to the aqueous subphase reduces the surface pressure of the compression isotherm and shifts it towards larger surfaces per molecule compared to the isotherm of azolectin alone. The compression isotherms for individual systems enable the determination of the numerous parameters characterizing Langmuir monolayers. Plotting the dependence of compressibility factor versus the area per molecule allows the determination of its maximum, which indicates the ordering of the molecule in the tested monolayer at the water/air interface. The maximum value of the compressibility factor for all the systems shown in the figure is within the range of 60-80 mN/m (Figure 1B, Table 1). The highest compressibility coefficient 72.5 mN/m was recorded for a system composed of azolectin and P4. The lowest value of 63.1 mN/m was identified in a system formed of an azolectin monolayer doped with P6.

Other parameters and their values, such as A_lift-off_—the value of the area per molecule at which an increase in the surface pressure above zero was recorded, π_collapse_ and A_collapse_—the values of pressure and surface per molecule at which the monolayer collapsed, are presented in Table 1. The table also presents the parameter χ—the ratio of A_collapse_ to the A_lift-off_, as a measure of molecules’ compressibility and orientation changes during compression. Moreover, the a_LE/LC_ parameter was presented, which is the slope coefficient in the dynamic increase in surface pressure on the isotherm. The lower the value of this coefficient, the easier it is to rearrange molecules at the water/air interface and form a packed monolayer. The azolectin monolayer, and the azolectin monolayer with P4, were characterized by the lowest a_LE/LC_. Similar values were obtained for the azolectin monolayer with P2, P5, and P6 compounds added in the subphase. For the monolayer composed of azolectin and compounds in the P2 and P6 subphases, the χ were the same.

### 2.2. Hysteresis of Compression/Decompression of π-A Azolectin, and the System of which it Is Composed, with P2, P4–P6 in the Water Subphase

**Figure 2:**
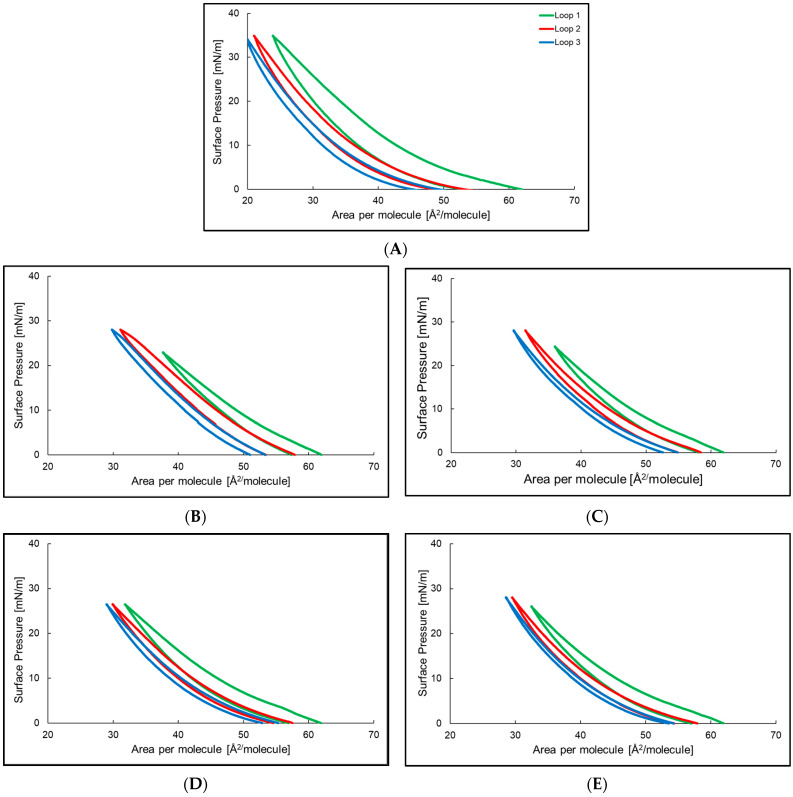
Hysteresis of the azolectin monolayer (**A**) and azolectin monolayer at the air/water interface at T = 25ºC with P2 (**B**), P4 (**C**), P5 (**D**), and P6 (**E**) in the water subphase. Loop 1 (─), loop 2 (─), and loop 3 (─).

Figure 2 presents the compression–decompression isotherms for the azolectin monolayer and the azolectin monolayer doped with 2, 4-6 peptide. In the pure azolectin system the hysteresis ranged between 0 and 35 mN/m. The rapid increase in surface pressure in the first cycle of compression was observed by 40 Å^2^ per molecule. The decompression plot does not follow the compression line, but occurs by lower surface area per molecule, with the highest surface pressure reached at about 20 Å^2^ per molecule. The hysteresis plot of azolectin with P2 ranged in surface pressure 0–28 mN/m. The surface pressure in the first compression cycle increased rapidly around 50 Å^2^ per molecule. The decompression plot did not follow the compression line, and occurred with a smaller surface area per molecule. The system behaved similarly in the second and third hysteresis loops. The hysteresis plot of the azolectin monolayers with P4, P5, and P6 had a similar course, except for small differences in the surface pressure when the systems reached the highest values of the parameter.

### 2.3. Monolayer Compression Isotherms of a Lecithin Monolayer with Peptides in the Aqueous Subphase

**Figure 3:**
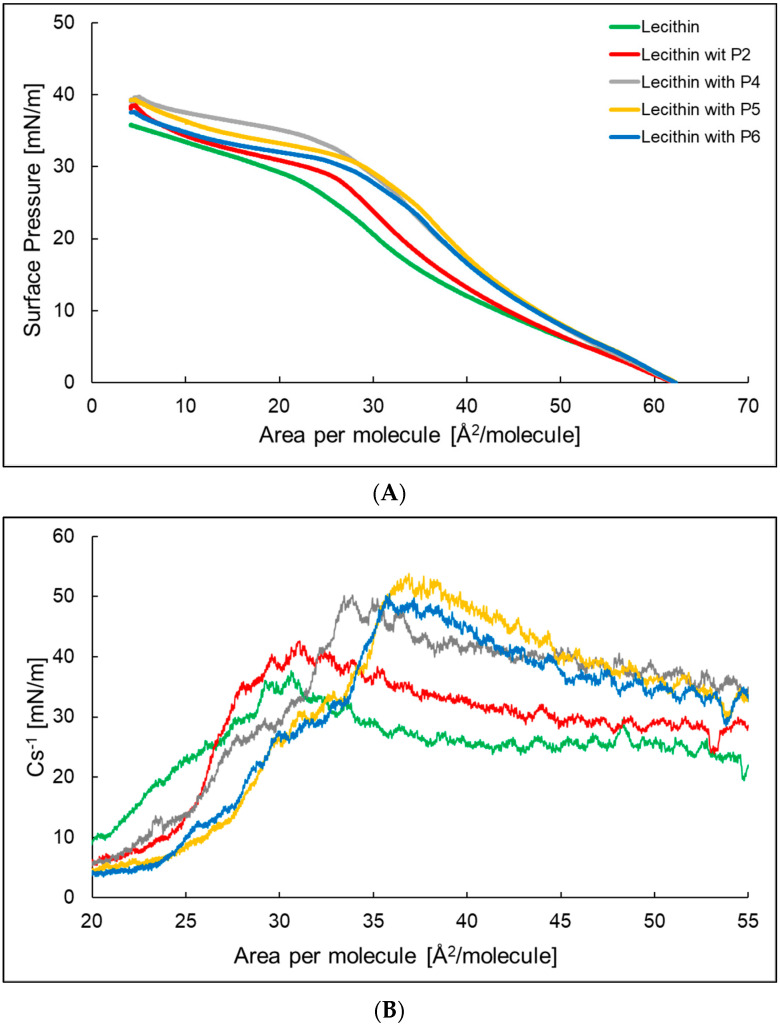
The compression isotherms (**A**) and dependence of the compressibility coefficient depending on the surface area per molecule (**B**) of the lecithin monolayer (─) at the air/water interface at T = 25 °C with P2 (─), P4 (─), P5 (─), and P6 (─) in the water subphase.

Figure 3A shows the compression isotherm of a lecithin monolayer and lecithin monolayer with P2, P4-P6. The surface area at which the surface pressure rises above 0 was, in all cases, about 62 Å^2^ per molecule. The lowest value of the surface pressure of the monolayer at which its collapse occurs was presented by lecithin. The addition of a peptide to the subphase increased the surface pressure at which the lecithin monolayer reaches a plateau and shifts it towards a higher Å^2^ per molecule. Figure 3B shows the compressibility factor for the lecithin monolayer and the lecithin monolayer with P2, P4–P6. The maximum compressibility factor was 37.7 and 42.6 mN/m, respectively, for the lecithin monolayer and lecithin monolayer with P2 (Table 2), indicating that the monolayers were in an expanded liquid state. In the case of the lecithin monolayer with P4, P5, and P6, the Cs^−1^_max_ value exceeded 50 mN/m, which proves that the formed monolayers were in the state of a condensed liquid. The a_LE/LC_ parameter for the lecithin monolayer showed the highest value, while for the lecithin monolayer with P2 and P4 it showed the lowest value. The addition of the peptides causes the molecules to rearrange more easily at the water/air interface to form a packed monolayer. In the case of the χ parameter, we observed an increase in the value after adding peptides to the subphase. For the lecithin monolayer with a peptide with more hydrophobic properties—P4 χ was the highest and equals 0.081. In the case of peptides with more hydrophilic properties, P2, P5, and P6, the χ values were similar.

### 2.4. Hysteresis of Compression/Decompression of π-A Lecithin, and the System of which it Is Composed, with P2, P4–P6 in the Water Subphase

**Figure 4:**
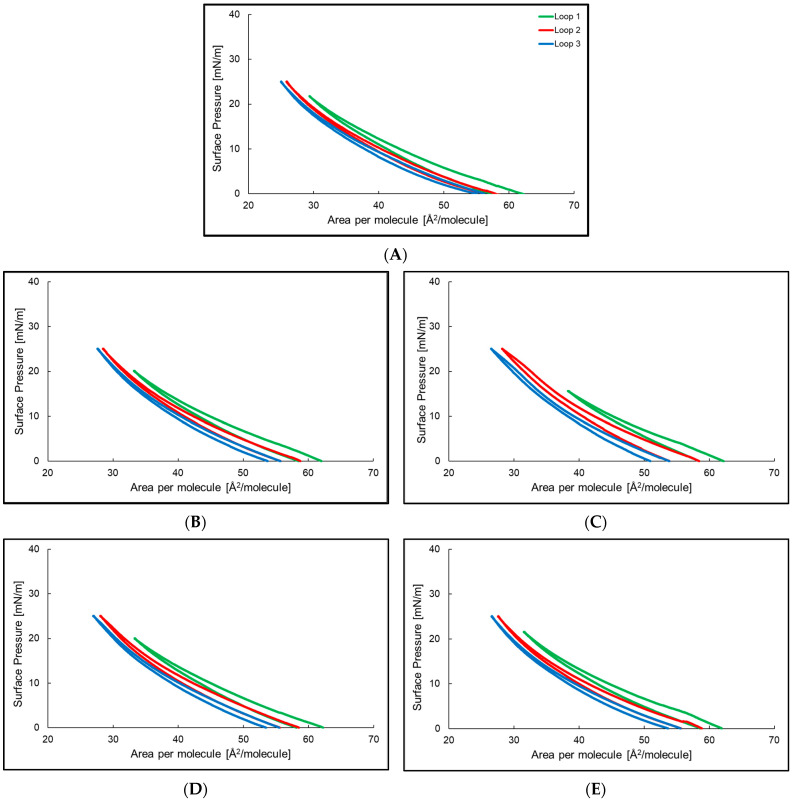
Hysteresis of the lecithin monolayer (**A**) and lecithin monolayer at the air/water interface at T = 25 °C with P2 (**B**), P4 (**C**), P5 (**D**), and P6 (**E**) in the water subphase. Loop 1 (─), loop 2 (─), and loop 3 (─).

Figure 4 shows the compression–decompression isotherms for the lecithin monolayer and P2, P4–P6 in the aqueous subphase. For the lecithin monolayer, the hysteresis was in the surface pressure range of 0–25 mN/m. The surface pressure for the first compression was beginning to increase rapidly at 50 Å^2^ per molecule. Decompression did not follow compression; it occurs with a smaller surface area per molecule. The highest measured value of surface pressure was reached at 25.0 Å^2^ per molecule. For the lecithin monolayer with P2, P4–P6 the hysteresis was in the same range of surface pressure as for the lecithin monolayer on the aqueous subphase without peptides. Comparing the hysteresis in Figure 4, there was a significant increase in the distance between the compression and decompression isotherms in the last loop for the lecithin monolayer with peptides in the aqueous subphase.

## 3. Discussion

Research conducted at the cellular level using living organisms and respective tissue samples is extremely complex and intricate. In contrast, the results obtained from such experiments may be ambiguous and difficult to explain. It is, therefore, justified to simplify the tested system in the first stage of the experiment, and then to modify it accordingly in order to verify the originally formulated research hypotheses. Langmuir’s single-layer technique is one of the most precise and simple methods for creating high-quality, ordered monolayers imitating bacterial membranes and their interactions with molecules in the subphase [25,26,27]. In the studies carried out above, a monolayer made of azolectin and lecithin was used as a model membrane. After adding P2, P4–P6 to the aqueous subphase, a decrease in the value of the surface pressure at which the plateau begins, and a shift of the curve towards larger surfaces per molecule, were observed on the compression isotherm of the azolectin monolayer. This may indicate a faster start of the phase transition after adding the peptides to the aqueous subphase [28,29,30]. On the other hand, the slope coefficient (a_LE/LC_) in the range of the dynamic increase in the surface pressure of the π-A isotherm of the azolectin monolayer increases after the addition of the tested peptides. This suggests a more difficult rearrangement of molecules at the water/air interface. The ability of the molecules to change their physical state in the monolayer determines the compressibility coefficient, which, for these systems, has a value of 60–80 mN/m, which indicates that the monolayer molecules are in the condensed liquid phase (LC) [31]. The addition of P4 to the subphase results in a compressibility coefficient increase and the addition of P2, P5, and P6 results in its reduction, compared to the system without the addition of peptides. The P4 compound has two hydrocarbon chains with twelve carbon atoms in its structure, which are hydrophobic, and the small polar head may interact stronger with the monolayer in this system, compared to water molecules or P4 molecules, creating a more ordered structure [28]. The azolectin monolayer, after adding P4, had a higher Cs-1max value than that with P5, which indicated that it is more fluid.

**Figure 5:**
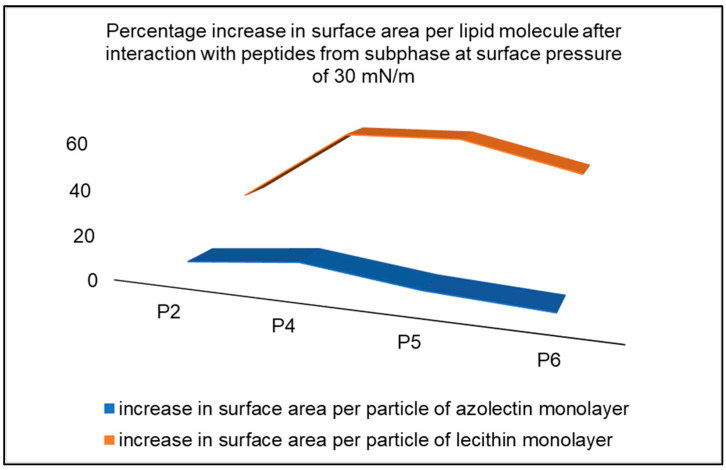
Percentage increase in surface area per lipid molecule after interaction with peptides from subphase, at surface pressure of 30 mN/m, with the azolectin and lecithin monolayer.

The interactions of peptides with a monolayer made of lecithin were quantitively different when compared to azolectin. In the graph of compression isotherms, we can see that the addition of peptides increases the pressure at which the lecithin monolayer reaches a plateau. This may indicate greater interactions between molecules in the lecithin monolayer than interactions between molecules in the water subphase, due to the surface pressure equation. The isotherms of lecithin monolayer are shifted towards higher surfaces per molecule, which, as in the case of systems with an azolectin monolayer, may indicate the incorporation of peptides into the monolayer [32]. The percentage increase in the surface area per lipid molecule, after interaction with the P2, P4–P6 peptides from the subphase, at a surface pressure of 30 mN/m, to the azolectin and lecithin monolayer, is shown in Figure 5.

The low slope coefficient a_LE/LC_ indicates an easier rearrangement in terms of the dynamic increase in surface pressure for these systems than for the monolayer formed of lecithin without peptides in the aqueous subphase. The reason may be the increase in the distance between the monolayer molecules caused by the penetration of the peptides. The a_LE/LC_ value is the highest for the system with P2 and the lowest with P5. This difference may be due to the fact that the P2 structure was characterized by the presence of intertwined amino acids lysine and tryptophan and it penetrated the water subphase deeper than any other system tested and should be considered the most hydrophilic of the systems tested, i.e., it may have little effect on the lecithin monolayer. In this case, the value of a_LE/LC_ would result mainly from the interactions occurring between the molecules of the lecithin monolayer. This is also confirmed by the compression isotherm plot for the monolayer formed of lecithin and P2 in the aqueous subphase, which is closest to the isotherm of the lecithin monolayer [33,34].

**Figure 6:**
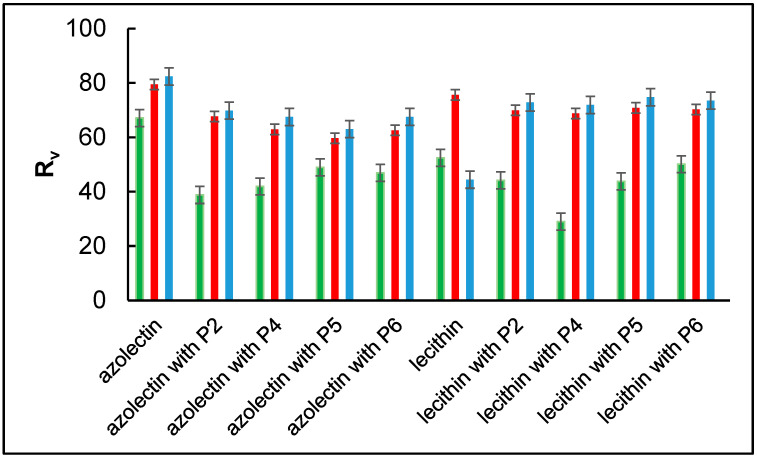
Isotherm compression reversibility coefficient R*_v_* for loop 1 (─), loop 2 (─), and loop 3 (─) of azolectin and lecithin monolayers with P2, P4, P5, and P6 peptides in the aqueous subphase. The error bars represent the SE standard error.

Table 3 and the Figure 6 show the results of the isotherm compression reversibility factor. Comparing all the tested systems, the highest value of 67.10% for loop 1 represents the azolectin monolayer and the lowest, 28.99%, represents the lecithin monolayer with P4 in the aqueous subphase. Thus, the value of R*_v_* in the first case indicates the greatest reversibility of the compression process, which may be due to the elastic or reversible interactions of the azolectin molecules. The lower reversibility of the films reflects the slower reorganization and spreading of the surface films compared with the rate of area expansion.

The isothermal compression reversibility coefficient in all systems, except for the monolayer made of lecithin, increased in successive loops of hysteresis, which supports the idea that the interactions between monolayer particles are stabilized. The shifts of hysteresis loops 2 and 3 towards smaller areas may be ascribed to the transition of molecules to the aqueous subphase [35]. The surface pressures at the defined molecule area decreased systematically in the following cycles, both in the case of azolectin and lecithin, which may also suggest the release of particles to the subphase or stronger interactions between the monolayer particles, e.g., intercalation.

According to Figure 6, the P4 caused some differences in lecithin monolayers, so it could be used in further research towards pharmaceutical applications. In the case of azolectin P2 and P4, we did not observe any significant difference. Taking into account all the results of our research, P4 would be the best candidate for further research, as it has a broad spectrum of activity against Gram-positive and Gram-negative bacteria, and antifungal activity against (Candida albicans, Candida tropicalis, and Aspergillus Niger). However, substances with medium lipophilicity, exhibiting high solubility in both water and lipids, will have optimal penetration ability, with a maximum at a logP value of 1.0–3.0. These requirements would be met by P2.

In the study of the effect of peptides (WKWK)_2_-KWKWK-NH_2_, (C12)_2_-KKKK-NH_2_, (KWK)_2_-KWWW-NH_2_, and (KK)_2_-KWWW-NH_2_ on a model bacterial membrane made of azolectin or lecithin, we observed differences in physicochemical properties depending on the molecules present in the evaluated systems. The design of AMPs analogs is aimed to increase their topical antimicrobial activity, while excluding the adverse effects of future dermatological pharmacotherapy. Applied peptide optimization strategies include, i.a., the structural modification of amino acids, development of hybrid peptides, and design of structurally and functionally related compounds [36], which are revealed in the present study and in other researchers’ studies.

## 4. Materials and Methods

### 4.1. Synthesis and Characterization of the Peptides

The preparation, purification, and determination of the structure of the peptides was carried out as described in our earlier publication [31] and is presented below.

Obtaining of peptide compounds

The Rink amide AM resin and the amino acids Fmoc-Lys(Boc)-OH, Fmoc-Lys(Fmoc)-OH, Fmoc-Arg(Pbf)-OH, and Fmoc-Trp(Boc)-OH were obtained from Iris Biotech (Germany). The dodecanoic acid, coupling reagents, and solvents used, such as N,N-dimethyl formamide (DMF), dichloromethane (DCM), 1-hydroxybenzotriazole (HOBt), trifluoroacetic acid (TFA), and acetonitrile (ACN), are from Merck (Germany).

The peptide sequences were de novo designed to present positive charge by the incorporation of lysine residues. Tryptophan residues and dodecanoic fatty acid were used to provide the ability to insert into bacterial membranes. The peptide compounds were manually synthesized using Fmoc solid phase peptide synthesis using the Rink amide AM resin (100–200 mesh; loading 0.48 mmol/g). The coupling reaction of the amino acids was made with the activators DIC and HOBt with three times molar excess of each amino acid and activator, dissolved in DMF/DCM (1:1; *v*/*v*) mixture. Deprotection was carried out with 20% (*v*/*v*) of piperidine in DMF. The de-anchoring of the peptides from the resin was achieved with TFA/TIS/H2O mixture in a volume ratio (95:2.5:2.5).

Lipopeptides characterization

The purity of the lipopeptides was analyzed using reverse phase high performance liquid chromatography (RP-HPLC) in an Shimadzu Nexera chromatograph with a DAD detector at 214 nm fitted with a Eurospher (100 × 4.6 mm) columns (Knauer, Berlin, Germany) using ACN:TFA (0.1%) and H2O:TFA (0.1%) as the mobile phase. The identity of lipopeptides was verified using matrix-assisted laser desorption time-of-flight (MALDI-TOF) spectrometry on MALDI-TOF/TOF 5800 (Sciex, Illinois, USA). Peptides with confirmed identities were freeze-dried (Christ, Hannover, Germany) and stored as dry powder at −20 °C.

Four of the six formerly evaluated peptides, were selected for further research: P2 (WKWK)_2_-KWKWK-NH_2_, P4 (C12)_2_-KKKK-NH_2_, P5 (KWK)_2_-KWWW- NH_2_, and P6 (KK)_2_-KWWW-NH_2._

### 4.2. Langmuir Films

The Langmuir–Wilhelmy trough, manufactured by Kibron Microtrough X (Helsinki, Finland), with the attached computer software Filmware X 4.0, were used to study the monolayers formed of azolectin or lecithin on the aqueous subphase with P2, P4–P6. The balance consisted of a tetrafluoroethylene (Teflon) 23.7 cm long and 7.9 cm wide tray, two movable Teflon barriers, and a platinum wire, used in place of a Wilhelmy plate, of 48.2 mg in weight and 0.5 mm in diameter, which ensured a negligible contact angle during the measurement. In order to avoid the introduction of impurities, before each measurement, the wire was rinsed with methanol, then with water and ignited in a burner flame. The barriers moved 10 mm per minute. A constant temperature of 25 °C ± 1 °C of the carrier phase was ensured using a thermostat. The cleanliness of the carrier phase surface was checked by measuring the surface tension. If the value of the voltage change did not exceed 0.30 mN/m during the movement of the railings towards the center of the tank, the surface was considered free of impurities. Otherwise, the washing procedure was repeated. The tubs were placed on anti-vibration tables. All measurements were performed in three repetitions.

### 4.3. Compression Isotherms of Azolectin or Lecithin on the Aqueous Subphase with P2, P4–P6

After checking the purity of the subphase, 15 μL of azolectin (Merck, Darmstadt, Germany) or lecithin (Merck, Darmstadt, Germany) solution (2.2 × 10^−3^ mol/L) in chloroform (Merck, Darmstadt, Germany) was applied. After the evaporation of the solvent, the monolayer was symmetrically compressed to a surface pressure of 5 mN/m, and then 15 μL of a 1.3 × 10^−3^ mol/L aqueous solution of the tested peptide was injected into the subphase. After 15 min of system stabilization, the barriers were slid off inwards at 10.02 mm/min. The force acting on the wire is expressed using the following formula [37]:

$$F={⍴}_{g}g{\pi r}^{2}l+2\gamma \left(\pi r\right)cos\theta -{⍴}_{l}{\pi r}^{2}h$$

where *F*—net force [N], ⍴*_g_*—wire density [$\frac{kg}{m^{3}}$], ⍴*_l_*—the density of the subphase [$\frac{kg}{m^{3}}$], *g*- gravitational constant [$\frac{N}{kg}$], *r*—wire radius [m], *l*—wire length [m], *h*—insertion depth of the wire [m], *γ*—the surface tension of the liquid [mN/m], and *θ*—contact angle.

The choice of azolectin and lecithin were dictated by the presence of negatively charged lipids, which are also the main type of bacterial membrane lipid. Thus, they may enable the testing of broad-spectrum antimicrobial peptides.

### 4.4. Hysteresis

Three compression and decompression cycles at 10 mm/min were applied to reveal the hysteresis loops. The measurements range was determined on the basis of isotherm courses in the FilmwareX 4.0 program. Using the formula proposed by Georgiev et al. [38], the isotherm compression reversibility factor was calculated using the following formula:

$$Rv=\frac{{\left(\int_{A_{collapse}}^{A_{lift-off}}\pi dA\right)}_{expansion}}{{\left(\int_{A_{collapse}}^{A_{lift-off}}\pi dA\right)}_{comprension}}\cdot 100\%$$

where *R_v_*—compression reversibility factor, *A_lift-off_*—lift-off area of surface pressure, and *A_collapse_*—area corresponding to the monolayer collapse.

### 4.5. Compressibility Coefficient of the Monolayer

The monolayer compressibility coefficient reflects the mechanical properties of the monolayer, and was calculated according to the following formula:

$$C_{S}^{-1}=-A\left(\frac{d\pi }{dA}\right)$$

where *C_S_*^−1^—compressibility factor [mN/m], *A*—surface area per molecule [Å^2^/molecule], and π—surface pressure [mN/m].

## 5. Conclusions

Studies have shown that the peptides (WKWK)_2_-KWKWK-NH_2_, (C12)_2_-KKKK-NH_2_, (KWK)_2_-KWWW-NH_2_, and (KK)_2_-KWWW-NH_2_ affect the organization of monolayer molecules, both those made of azolectin and of lecithin, at the liquid/air interface. They cause a change in their surface properties, depending on the structure of peptide implemented into the monolayer, as well as on the type of monolayer itself. The addition of peptides to the aqueous subphase shifts the phase transition in the monolayers towards smaller areas per molecule in all cases. This is equivalent to layer compression and leads to a denser packing of molecules on the surface of the subphase. A clear differentiation between the compression isotherms of the azolectin and lecithin monolayers was demonstrated. In the first case, we noticed a decrease in the plateau pressure after the addition of peptides, while, in the second, we noticed an increase. This is due to the fact that the addition of peptides diminishes the tension of the azolectin monolayer and increases the tension of the lecithin monolayer. The surface pressure parameter, compressibility, and compression reversibility of the monolayer can be used to rationally design antimicrobial agents with selective toxicity to specific organisms.

## Figures and Tables

**Table 1:** Characteristic parameters of π–A isotherms: A_lift-off_—lift-off area of surface pressure. A_collapse_—area corresponding to the monolayer collapse, π_collapse_—collapse pressure [mN/m], Cs^−1^ _max_ —maximum value of the compression modulus [mN/m] (refers to A_max_ or π_max_), χ (A_collapse_/A_lift-off_), a_LE/LC_—slope coefficient in the range of the dynamic increase in the surface pressure of the π–A isotherm.

	A_lift-off_ (Å^2^/molec.)	A_collapse_ (Å^2^/molec.)	π_collapse_ (mN/m)	A_max_ (Å^2^/molec.)	π_max_ (mN/m)	C_s_^−1^_max_ (mN/m)	χ	a_LE/LC_
Azolectin	61.6	4.2	45.0	35.8	24.5	68.4	0.068	−1.79
Azolectin with P2	61.9	4.2	40.9	20.0	42.1	64.3	0.068	−1.30
Azolectin with P4	61.6	4.4	40.2	38.0	27.8	72.5	0.071	−1.53
Azolectin with P5	61.8	4.8	42.0	42.5	19.2	64.7	0.078	−1.33
Azolectin with P6	61.7	4.2	41.6	38.9	22.6	63.1	0.068	−1.36

**Table 2:** Characteristic parameters of π–A isotherms: A_lift-off_—lift-off area of surface pressure. A_collapse_—area corresponding to the monolayer collapse, π_collapse_—collapse pressure [mN/m], Cs^−1^ _max_ —maximum value of the compression modulus [mN/m] (refers to A_max_ or π_max_), χ (A_collapse_/_Alift-off_), a_LE/LC_—slope coefficient in the range of the dynamic increase in the surface pressure of the π–A isotherm.

	A_lift-off_ (Å^2^/molec.)	A_collapse_ (Å^2^/molec.)	π_collapse_ (mN/m)	A_max_ (Å^2^/molec.)	π_max_ (mN/m)	C_s_^−1^_max_ (mN/m)	χ	a_LE/LC_
Lecithin	62.0	4.2	35.8	30.6	19.9	37.7	0.068	−0.673
Lecithin with P2	61.8	4.6	38.6	31.0	22.4	42.6	0.074	−0.765
Lecithin with P4	62.0	5.0	39.8	33.9	24.2	50.2	0.081	−0.924
Lecithin with P5	62.2	4.5	39.3	36.9	21.5	53.8	0.072	−0.929
Lecithin with P6	62	4.5	37.6	35.8	21.6	50.5	0.073	−0.879

**Table 3:** Isotherm compression reversibility coefficient R*_v_* for loops 1-3 of azolectin and lecithin monolayers with P2, P4, P5, and P6 peptides in the aqueous subphase.

R_v_	Azolectin	Azolectin with P2	Azolectin with P4	Azolectin with P5	Azolectin with P6	Lecithin	Lecithin with P2	Lecithin with P4	Lecithin with P5	Lecithin with P6
loop 1	67.10	38.79	41.91	48.96	46.89	52.42	44.15	28.99	43.79	50.09
loop 2	79.44	67.68	62.92	59.66	62.58	75.62	69.94	68.76	70.83	70.23
loop 3	82.39	69.82	67.46	63.03	67.53	44.45	72.81	71.92	74.74	73.50

## Data Availability

The scientific data are available at Wroclaw Medical University, Department of Physical Chemistry and Biophysics.
